# Giant cell tumors of the mobile spine with invasion of adjacent vertebrae: an unusual imaging finding

**DOI:** 10.1186/s12891-021-04610-0

**Published:** 2021-08-24

**Authors:** Gang Jiang, Ling-Ling Sun, Yong-Jun Ye, Zhi-Tao Yang, Qing-lian Ji, Jing Pang, Chuan-Ping Gao

**Affiliations:** 1 Department of Radiology, The affiliated hospital of Qingdao University, Qingdao, 266003 China; 2 Department of Pathology, The affiliated hospital of Qingdao University, Qingdao, 266003 China; 3 Department of Radiology, Lishui Hospital of Zhejiang University, Lishui, 323000 China

**Keywords:** Giant cell tumors, Mobile spine, Adjacent vertebrae invasion, Computed tomography, Magnetic resonance imaging

## Abstract

**Background:**

Giant cell tumors of the mobile spine invasion of the adjacent vertebrae are an ignored imaging finding.

**Methods:**

Nine patients with giant cell tumors of the mobile spine with invasion of the adjacent vertebrae confirmed by pathology were enrolled. Eight patients had pure giant cell tumors (GCTs), while one patient also had an aneurysmal bone cyst. All patients underwent conventional computed tomography, three-dimensional reconstruction, and conventional magnetic resonance imaging, while seven patients also underwent post-contrast magnetic resonance imaging.

**Results:**

All patients showed GCTs of the mobile spine that arose from the vertebral body and extended to the vertebral arch. The tumors showed soft-tissue attenuation with no evidence of a mineralized matrix. Pathological fracture was seen in five patients. The margin of the original tumor showed partial sclerosis in four patients and involved an adjacent vertebral body with a sclerotic rim in two patients. The tumors showed a homogeneous and similar signal intensity to the normal spinal cord on T1WI (T1-weighted image) in five patients. The cystic area of the tumors was hyperintense on T2WI in the remaining four patients, while one patient showed hemorrhage that was hyperintense on T1WI. The solid components of the GCTs show marked enhancement in all cases, while the cystic area of the tumors was observed without enhancement on contrast-enhanced images in four patients. Bone destruction of the adjacent vertebral body showed a homogeneous signal on T1WI and T2WI and marked enhancement on contrast-enhanced images.

**Conclusions:**

Giant cell tumors of the mobile spine with invasion into adjacent vertebrae are an unusual imaging finding. Radiologists should be familiar with this imaging characteristic.

## Background

Giant cell tumors (GCTs) of the bone are relatively rare and account for 5% of all primary bone tumors [1]. They predominantly occur in the long bones near articulations after skeletal maturity, especially around the knee joint. However, GCTs occur infrequently in the spine, with a predominant sacral location, while their presence in the mobile spine above the sacrum is even rarer. GCTs of the spine occurring at the sacral spine typically invade multiple segments, while GCTs occurring at the mobile spine are confined to one vertebra. GCTs are benign but exhibit very aggressive local behavior. The majority of the mobile spine GCTs arising from the vertebral body and extending into the vertebral arch. However, GCTs of the mobile spine that involve adjacent vertebrae are extremely rare, with few reports in the literature [2, 3]. Herein, we report nine cases of GCTs of the mobile spine that invaded the adjacent vertebrae.

## Methods

This study was approved by our hospital ethics committee with all the patients given their signed informed consent. We performed a retrospective analysis of cases with GCTs of the mobile spine that invaded the adjacent vertebrae between 2011 and 2019. Nine patients (seven men, two women; mean age = 33 years [range, 19–62 years]) were included. The radiological images of all patients were analyzed. The study was approved by the ethics committee of the authors’ institution.

Patients were evaluated by computed tomography (CT) (LightSpeed; General Electric Medical Systems, Milwaukee, WI, USA) and magnetic resonance imaging (MRI) (General Electric Medical Systems) before surgery. Available clinical data included age at presentation, sex,medical history, and the duration of symptoms. CT examination using 16-slice multi detector spiral CT systems was performed in nine patients. A 3- or 5-mm section thickness was used for diagnostic reading and the reconstruction interval. Image data were reformatted in all cases for viewing in the sagittal plane. MRI was performed with a 1.5 T Signa HD MR unit. Sagittal T1-weighted images (repetition time = 460–600 ms, echo time = 7–20 ms, 4 excitations), sagittal and transverse T2-weighted images (repetition time = 3000–4700 ms, echo time = 88–128 ms, 4–6 excitations), and sagittal T2-weighted fat-suppression images were obtained. Contrast-enhanced images in the axial, sagittal, and coronal planes were obtained using a T1-weighted spin-echo sequence with fat suppression for six of the nine patients.

Two experienced musculoskeletal radiologists evaluated the imaging independently and the results were determined by consensus. The CT images were analyzed for bony destruction and minor bone changes. The MR images were also evaluated for signal intensities and the degree of contrast enhancement. Invasion of adjacent vertebrae was assessed on reformatted sagittal plane CT and MR images.

## Results

### Location of the lesions

The spine GCT usually affects the vertebral body and this original site is primary part of GCT. Spine GCT can be locally aggressive and extend to adjacent vertebral segment that called secondary part of GCT. The original location of the mobile spine GCTs were the cervical spine in two patients, thoracic spine in six patients, and lumbar spine in one patient. Secondary invasion of the upper vertebrae was observed in four patients, while secondary invasion of the lower vertebrae was observed in five patients. The secondary-involved vertebral body was located anterior in three patients and posterior in six patients (Table 1).

**Table 1:** Clinical finding of nine patients with giant cell tumors of the mobile spine involving the adjacent vertebrae

Case	Age(Year)	Symptoms	Original location	location of adjacent vertebrae involved	Duration of Symptoms(months)
	Sex				
1	27/M	Paralysis of both lower extremities	T12 body	Anterior part of L1 body	1/3
2	32/M	Back pain and dyspnea	T12 body	Posterior part of T11 body	6
3	23/M	Cervical pain with weakness of extremities	C4 body	Posterior part of C3body	2
4	32/M	Upper back pain	T2 body	Anterior part of T3 body	2
5	62/F	Middle back pain	L1 body	Posterior part of L2 body	6
6	25/F	Upper back pain	T5 body	Posterior part of T6 body	3
7	19/M	Upper back pain	T2 body	Posterior part of T3 body	4
8	42/M	Middle back pain and weakness of the right lower extremity	T10 body	Posterior part of T9 body	7
9	35/M	Cervical pain and dyspnea	C7 body	Anterior part of T1 body	5

### CT images

**Fig. 1:**
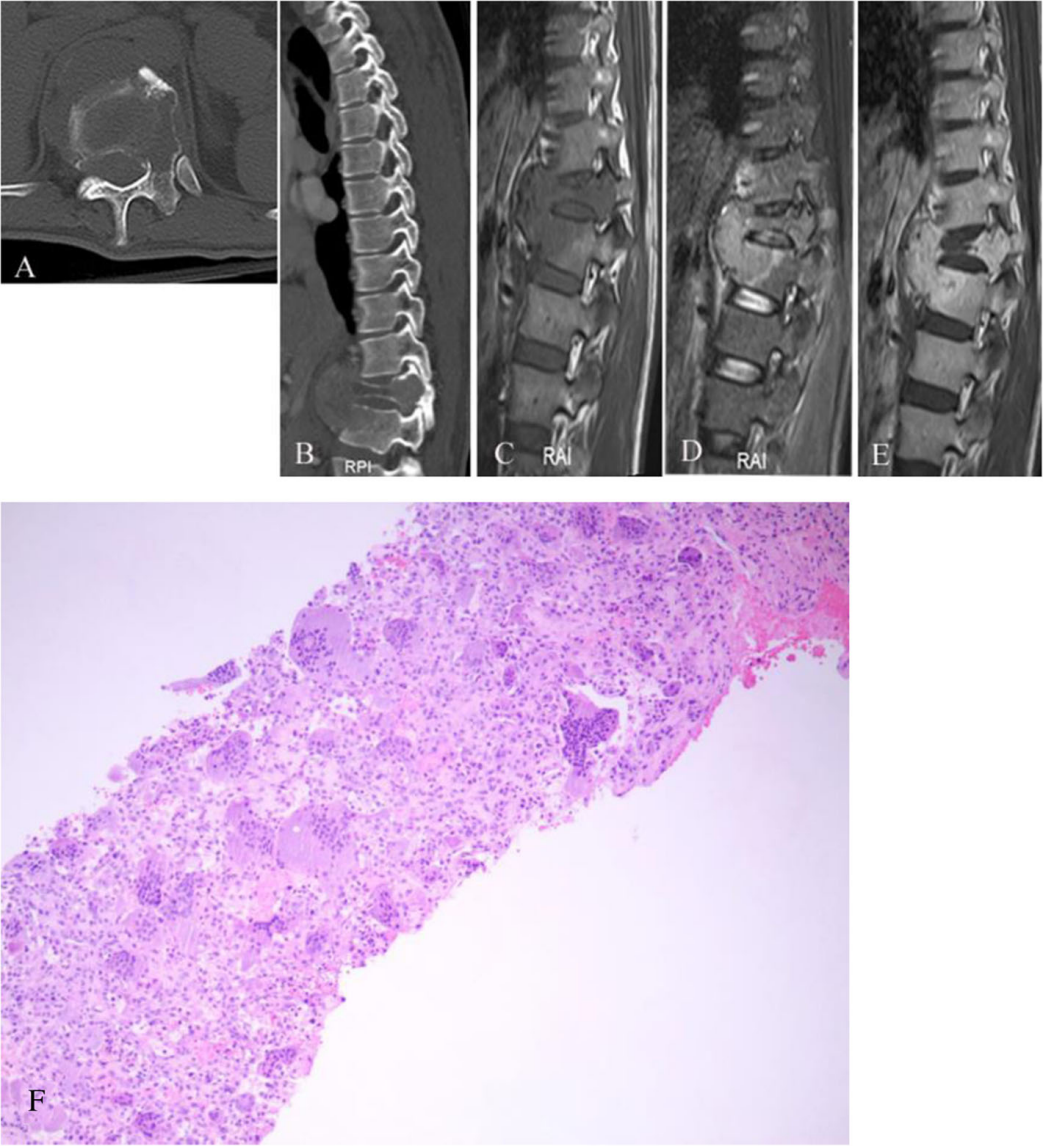
Giant cell tumor originating in the Th12 vertebral body. **a** Axial computed tomography (CT), **b** reformatted sagittal plane, **c** sagittal plane T1-weighted image, **d** sagittal plane T2-weighted image, and **e** post-enhanced sagittal T1-weighted image showing lytic bony destruction in the Th12 vertebral body. (**a**) The lesion invaded the left pedicle and the margin was ill-defined. **b–e** The vertebral body had collapsed and the soft tissue of the anterior vertebral body had invaded the anterosuperior L1 vertebral body. **e** The lesion showed significant enhancement on contrast-enhanced magnetic resonance imaging (MRI). **f** Photomicrograph of the tissue from patient 1. Hematoxylin and eosin (H&E)-stained tissue section (200× magnification) demonstrated a highly cellular, solid neoplasm consisting of mononuclear cells as well as osteoclast-like giant cells

**Fig. 2:**
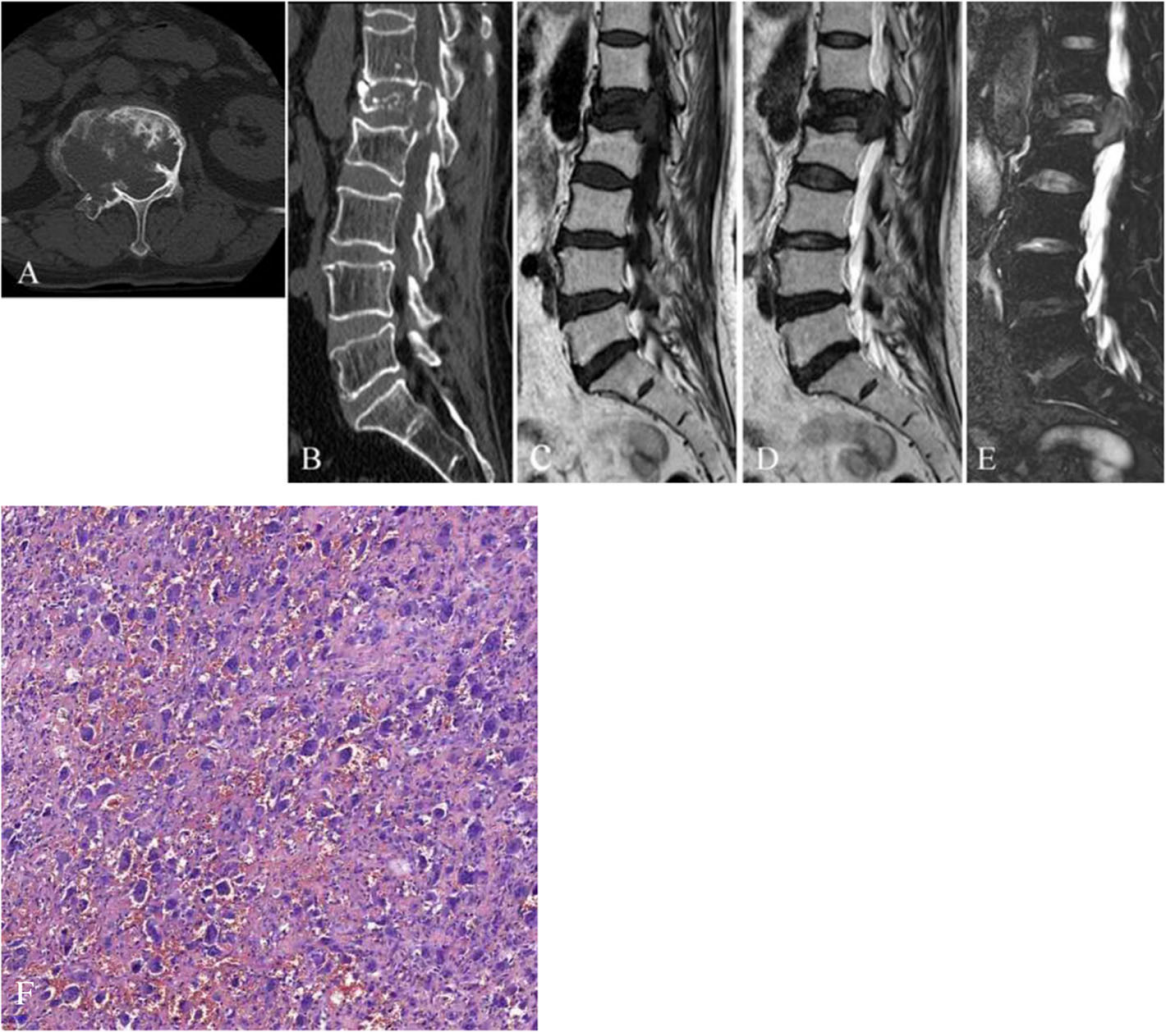
Giant cell tumor originating from the L1 vertebral body. **a** Axial computed tomography (CT), **b** reformatted sagittal plane, **c** sagittal plane T1-weighted image, **d** sagittal plane T2-weighted image, and **e** sagittal short time inversion recovery (STIR) image. **a** The lesion invaded the left pedicle with marginal sclerosis and invaded the right transverse process with an ill-defined margin. **b** The lesion invaded the anteroposterior L1 vertebral body with a well-defined margin. The lesion showed an intermediate signal intensity in T2-weighted image (T2WI) and STIR, while the mass compressed the dural sac and invaded the L1 vertebral body. (**f**) Photomicrograph (200× magnification) demonstrating highly giant cells, as well as osteoclast-like giant cells with a uniform distribution

**Fig. 3:**
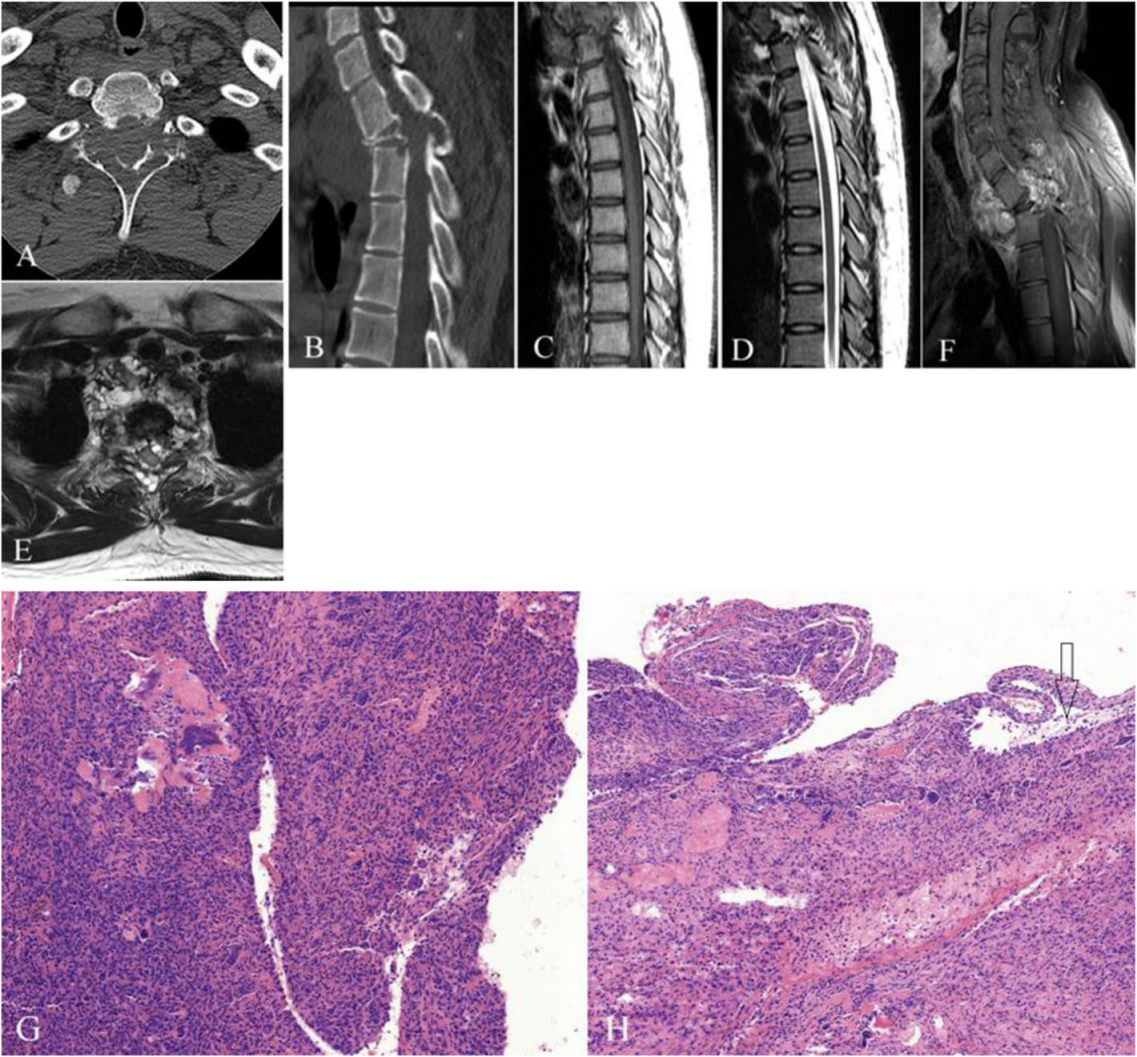
Giant cell tumor in the T2 vertebra. **a** Axial CT, **b** reformatted sagittal plane image, **c** sagittal plane T1-weighted image, **d** sagittal plane T2-weighted image, **e** axial T2-weighted image, and **f** post-enhanced sagittal and T1-weighted image. **a** The lesion involved the bilateral lamina and the spinous process with an ill-defined margin. **b** T2 vertebral body collapse and lesion invasion into the anteroposterior T3 vertebra. **c–e** MRI showing multiple cystic components and fluid–fluid levels that compressed the dural sac. **f** The solid component showed significant enhancement on post-enhanced image. Photomicrograph (100× magnification) demonstrating (**g)** a multinuclear giant cell and (**h**, arrow) an aneurysmal bone cyst component showing cyst-like changes, intracavitary bleeding, and no endothelial cells in the wall

All nine patients showed GCTs arising from the vertebral body. The tumors showed soft-tissue attenuation with no evidence of mineralized matrix. The left pedicle of the vertebral arch was involved in three patients (Fig. 1A); the right pedicle of the vertebral arch was involved in one patient (Fig. 2A); the right pedicle and lamina of the vertebral arch were invaded in three patients; the bilateral pedicle of the vertebral arch was invaded in one patient; and the bilateral pedicle, lamina, and spinous process of the vertebral arch were invaded in one patient (Fig. 3A). A pathological fracture was seen in five patients (Fig. 1B-E), while the vertebral body was presented collapse in one of five patients (Fig. 3A). The original vertebrae lesion, with partial margin sclerosis, was well presented in four patients. An invaded vertebrae with a sclerotic margin was observed in patients 5 and 6. Detection of adjacent vertebrae invasion was most sensitive in the sagittal plane on CT and MRI images, especially when there was only minor bone destruction (Fig. 2B, and 3B).

### MRI

There were eight cases of pure GCTs and one case of a GCT associated with a secondary aneurysmal bone cyst (ABC). A summary and illustration of the MRI features are shown in Table 2 and Figs. 1, 2 and 3. T1-weighted MRI showed a homogeneous and similar signal intensity to the normal spinal cord in five patients, while the solid components of the remaining four patients showed a heterogeneous signal intensity. Of these patients, the cystic area had a low signal intensity in three patients and a low signal cystic area and a high signal intensity representing hemorrhage in one patient (patient 7). T2-weighted MRI showed an intermediate or hyperintense signal in five patients, while the cystic area and hemorrhage area showed a high signal intensity in the remaining four patients. The solid components of the GCTs showed marked enhancement on contrast-enhanced MRI in all patients. Cystic areas without enhancement were observed on contrast-enhanced images, particularly in patient 7, who also had an ABC. The components of invaded adjacent vertebrae showed a homogeneous signal in T1WI and T2WI because there was no cystic or hemorrhagic injury in our series.

**Table 2:** Imaging characteristics of nine patients with giant cell tumors of the mobile spine involving the adjacent vertebrae

Case	Original location	Fractures or collapse	T1WI	T2WI	Enhancement	Margin sclerosis	Cortical destruction	Margin of involving adjacent vertebrae	Enneking Stage
1	T12	+	isointense	hyperintense	significant	_	+	ill-defined	3
2	T12	–	heterogeneous iso-low	heterogeneous polycystic high	heterogeneous high-low	+partial	+	ill-defined	3
3	C4	+	isointense	hyperintense	significant	_	+	ill-defined	3
4	T2	–	heterogeneous iso-low	heterogeous polycystic high	heterogeneous high-low	_	+	ill-defined	3
5	L1	+	hypointense	intermediate	null	+partial	+	well-defined	3
6	T5	–	isointense	intermediate	null	+partial	+	well-defined	3
7^a^	T2	+	Heterogeneous iso-low	Heterogeneous with polycystic high	Heterogeneous with polycystic high	–	+	ill-defined	3
8	T10	–	hypointense	intermediate	significant	+partial	+	ill-defined	3
9	C7	+	Heterogeneous iso-high	Heterogeneous low-iso-high	Heterogeneous low-iso-high	–	+	ill-defined	3

+, present; −, absent; ^a^ GCT associated with an aneurysmal bone cyst

### Treatment

Surgical resection was performed in all patients. Subtotal resection was performed in case 7 and the lesion originates from T2 associated with aneurysmal bone cyst. *En bloc* spondylectomy was undertaken in eight patients of pure giant cell tumors. After surgery, their pain significantly alleviated in patients with *En bloc* resection. The case with subtotal resection suffered residual disease in 13-months follow-up.

## Discussion

GCT of the bone was first described by Sir Astley Cooper in 1818 [4]. The most common sites of presentation are the long bones, followed by the pelvis. GCT of the spine is uncommon, accounting for approximately 16.2% of all primary spinal tumors [5]. The majority of these spinal lesions occur in the sacrum, followed by the thoracic, cervical, and lumbar segments in order of decreasing frequency [6]. Most case series indicate that GCTs are more common in women than in men, with ratios ranging from 1.1:1 to 1.5:1 [7]. GCTs usually occur in people aged 20–40 years old, with the peak prevalence in the third decade of life [8].

GCTs of the bone contain many giant cells with a diffuse distribution in a background of mononuclear cells. These mononuclear cells resemble normal histiocytes. The stroma of most GCTs is vascular and contains numerous thin-walled capillaries. These lesions may be associated with secondary ABC formation. The identification of these solid areas of GCTs allows their differentiation from primary ABCs.

GCTs invading the spine are rare. The majority of these tumors occur at the sacrum rather than at the spine above the sacrum, with sacral tumors accounting for 2–8% of GCTs [9]. GCTs that occur in the sacrum generally invade multiple segments. For example, Martin et al. [9] reported that the most frequent location was the upper two segments, although some lesions were so large that they invaded the entire sacrum. However, the majority of GCTs of the mobile spine are confined to one vertebra [7, 9] and originate in the vertebral body and extended to the vertebral arch. Demura et al. [10] reported a GCT case that originated from Th12 with lytic bony destruction in the vertebra and the right ribs, involving a soft tissue mass that compressed the right lung and spinal canal. Shi et al. [2] also reported a GCT case that originated from C7, with tumors invading the anterior vertebral body of C6 and T1. Because lytic lesions of the original GCTs are eccentric, paraspinal soft tissues mass are local prominent which causing adjacent vertebral body bony destruction. The present study describes nine cases of GCTs in the mobile spine that invaded the adjacent vertebrae, with invasion of the anterior vertebral body in three cases and the posterior vertebral body in six cases.

GCTs are histologically benign tumors, although they often take an aggressive clinical course. The cortex at the lytic areas becomes thin, gets penetrated, or disappears with an associated soft-tissue mass. If the lesion extends into the arch, bony destruction with an ill-defined margin can occur. Case studies have reported a low rate of sclerotic margins in GCTs (1–2% of lesions) [8, 11]. The sclerotic margin shows a thin sclerotic border from the surrounding normal bone CT and a band of low signal intensity on MRI, which corresponds to both a fibrous capsule and reactive sclerosis [12]. Aoki et al. [12] suggested that bony sclerosis is a host reaction against the tumor and correlates with a less aggressive radiographic appearance, which suggests that the tumors are slow-growing. In the present study, two patients showed a sclerotic margin of the invaded adjacent vertebral body, which suggests a long history in these patients as well as an aggressive pattern of the lesions. The pathological feature of the GCTs in our series was similar to common GCTs.

GCTs are the most common lesion associated with secondary ABCs and account for 39% of lesions [8, 13]. Murphey et al. [14] reported that GCTs with prominent ABC elements may present with a more aggressive radiographic appearance, which reflects the expansile cystic component. Imaging features of GCTs suggesting local aggressiveness include a large size, rapid growth, ill-defined boundaries, and invasion of the surrounding soft tissues [15]. MRI is also sensitive for detection of cystic components and fluid–fluid levels secondary to ABCs. The solid areas of GCTs show marked enhancement on contrast-enhanced CT and MRI because of the hypervascular nature of the tumor, which allows differentiation from primary ABCs. In patient 7 of our series, the vertebral body was weakened by diffuse bony destruction causing a compression fracture.

According to the Enneking classification for benign tumors [16], the GCTs of the mobile spine in our series belong to Enneking stage III. The patients treated with en bloc vertebrectomy had a lower recurrence rate. Nevertheless, en bloc resection cannot be applied in all patients because critical structures adjacent to the vertebrae, such as the pedicle and the accessory of the vertebrae, can be invaded by the tumor. Nevertheless, there are several promising adjuvant treatments for decreasing the recurrence of SGCT, including stereotactic radiotherapy, selective arterial embolization, and inactivation of the lesion site.

The most important differential diagnosis is tuberculosis spondylitis. The disease process begins in the anterior part of the vertebral body adjacent to the endplate. MRI finding may be endplate edema in early stage. Involvement of the disk manifests as collapse of the intervertebral disk space. With progression of disease, there is development of progressive vertebral collapse and wedging of multiple vertebral bodies leading to the characteristic angulation and gibbus deformity. Paravertebral abscesses form early and are easily seen in the thoracic region. Abscesses may spread through tissue planes to distant body parts. The psoas abscess may extend to the groin, thigh and hips [17, 18].

## Conclusions

Although rare, GCTs can occur in the mobile spine. GCTs in the mobile spine are typically confined to one vertebra, while those invading the adjacent vertebrae are extremely rare. Nevertheless, clinicians should recognize these uncommon imaging findings.

## Data Availability

All data and materials are available from the corresponding author on reasonable request.

## Abbreviations

GCTs: Giant cell tumors
ABC: Aneurysmal bone cyst
MRI: Magnetic resonance imaging
CT: Computed tomography
STIR: Short time inversion recovery
